# The 3D-Printed Bilayer’s Bioactive-Biomaterials Scaffold for Full-Thickness Articular Cartilage Defects Treatment

**DOI:** 10.3390/ma13153417

**Published:** 2020-08-03

**Authors:** Kittiya Thunsiri, Siwasit Pitjamit, Peraphan Pothacharoen, Dumnoensun Pruksakorn, Wasawat Nakkiew, Wassanai Wattanutchariya

**Affiliations:** 1 Doctoral Program in Biomedical Engineering, Biomedical Engineering Institute, Chiang Mai University, Chiang Mai 50200, Thailand; kittiya.thunsiri@hotmail.com; 2 Biomedical Engineering Institute, Chiang Mai University, Chiang Mai 50200, Thailand; dumnoensun@hotmail.com; 3 Advanced Manufacturing Technology Research Center (AMTech), Department of Industrial Engineering, Faculty of Engineering, Chiang Mai University, Chiang Mai 50200, Thailand; siwasit_p@cmu.ac.th (S.P.); wasawat@eng.cmu.ac.th (W.N.); 4 Graduate School, Chiang Mai University, Chiang Mai 50200, Thailand; 5 Thailand Excellence Center for Tissue Engineering and Stem Cell, Department of Biochemistry, Faculty of Medicine, Chiang Mai University, Chiang Mai 50200, Thailand; peraphan.pothacharoen@gmail.com; 6 Muscoloskeletal Science and Translational Research (MSTR) Center, Chiang Mai University, Chiang Mai 50200, Thailand; 7 Omics Center for Health Science, Faculty of Medicine, Chiang Mai University, Chiang Mai 50200, Thailand

**Keywords:** 3D printing, bilayer scaffold, biomaterials, tissue engineering, full-thickness articular cartilage defects

## Abstract

The full-thickness articular cartilage defect (FTAC) is an abnormally severe grade of articular cartilage (AC) injury. An osteochondral autograft transfer (OAT) is the recommended treatment, but the increasing morbidity rate from osteochondral plug harvesting is a limitation. Thus, the 3D-printed bilayer’s bioactive-biomaterials scaffold is of major interest. Polylactic acid (PLA) and polycaprolactone (PCL) were blended with hydroxyapatite (HA) for the 3D-printed bone layer of the bilayer’s bioactive-biomaterials scaffold (B-BBBS). Meanwhile, the blended PLA/PCL filament was 3D printed and combined with a chitosan (CS)/silk firoin (SF) using a lyophilization technique to fabricate the AC layer of the bilayer’s bioactive-biomaterials scaffold (AC-BBBS). Material characterization and mechanical and biological tests were performed. The fabrication process consists of combining the 3D-printed structure (AC-BBBS and B-BBBS) and a lyophilized porous AC-BBBS. The morphology and printing abilities were investigated, and biological tests were performed. Finite element analysis (FEA) was performed to predict the maximum load that the bilayer’s bioactive-biomaterials scaffold (BBBS) could carry. The presence of HA and CS/SF in the PLA/PCL structure increased cell proliferation. The FEA predicted the load carrying capacity to be up to 663.2 N. All tests indicated that it is possible for BBBS to be used in tissue engineering for AC and bone regeneration in FTAC treatment.

## 1. Introduction

Articular cartilage (AC) injury from chronic joint stress or acute traumatic injuries results in pain and swelling, causing long-term problems for patients [1,2,3,4]. The AC is devoid of blood vessels, lymphatics, and nerves, hence there is a limited capacity for intrinsic healing and repair [3,4,5,6]. According to the International Cartilage Repair Society (ICRS) cartilage lesion classification system, the most abnormally severe grade is classified when the lesion extends from the superficial of the AC to the subchondral bone (grade IV: full-thickness AC defects) [7,8]. Currently, the most promising technique to help full-thickness AC defect (FTAC) patients to resume their previous sporting activities is the osteochondral autograft transfer (OAT), in which the defect is filled with the same person’s osteochondral (OC) tissue taken from less weight-bearing areas on the femoral condyle. This can be in the form of either a single large OC plug or multiple small plugs, and it achieves a congruency of the AC surface in the load-bearing zone of the femoral condyle [6,9]. OAT implantation does not have the same limitations as other treatments in terms of time of rehabilitation and predictable cartilage-type results, and has become widely accepted as an effective treatment for high-grade AC defects of the knee and talus [10,11,12]. However, there are some limitations of OAT such as the increased morbidity rate at the donor site and high operation cost [6,13,14]. Thus, a biomedical engineering strategy was used to eliminate the limitations of the OAT technique for FTAC. The bilayer biomaterials scaffold and its fabrication process are of major interest to improve the treatment process and decrease the limitations using low-cost local materials and fabrication techniques. In our previous study, 3D printing and the fabricated biomaterials filaments were demonstrated as being feasible for use as an implantation device for bone fracture treatment [15]. Polylactic acid (PLA), polycaprolactone (PCL), and hydroxyapatite (HA) were selected to fabricate 3D-printed filaments owing to both their biological and mechanical abilities [15,16,17,18,19,20,21,22]. PLA is produced from 100% renewable resources and can be obtained from the fermentation of corn, sugar beets, and rice [23]. The USA Food and Drug Administration (FDA) approved PLA in the 1970s [24]. Since then, this polymer has been widely applied for medical purposes owing to its excellent biocompatibility, biodegradability, and mechanical properties [25,26,27,28]. PCL has been widely used in tissue engineering. The benefits of this polyester are its biocompatibility, relatively slow degradation rate, substantially fewer acidic breakdown products in comparison with other polyesters, and suitability for use in load-bearing applications [29,30,31]. It has also been reported to have an exceptional ability to form blends with a wide variety of polymers [32]. PCL is considered as a non-toxic and tissue-compatible polymer that is widely used for medical device fabrication, tissue engineering, and drug-release applications [33,34,35,36,37,38,39,40,41,42,43,44,45]. Modified PCL, using monomers with various functional groups, copolymerization, and post-functionalization to introduce functionality, demonstrates useful changes in its mechanical properties, hydrolytic degradation, and crystalline structures compared with native PCL [46,47,48,49,50]. HA can be obtained from natural sources such as mammalian bones, coral, and seashell [51,52,53,54]. HA is used in bone tissue engineering applications owing to its bioactivity, osteo-conductivity, and biodegradability [55,56]. HA powder can be added to other biomaterials to increase their biological properties [57,58]. Moreover, chitosan (CS) and silk fibroin (SF) were selected to be additional materials for AC layer fabrication. CS is a type of polysaccharide derived from chitin with a structure similar to the glycosaminoglycans (GAGs), which are a component of the cartilage extracellular matrix [12,59]. CS is widely used in tissue engineering applications owing to its porous structure, biocompatibility, biodegradability, non-cytotoxicity, and low cost [57,58,60]. Furthermore, implantation of the CS scaffold could reduce the risk of bacterial infection owing to its antimicrobial property [61,62,63]. Moreover, a previous study reported that the structure of CS could promote osteo-conductivity [64]. Meanwhile, silk fiber is recognized as a raw material for the textile industry and contains useful components (silk fibroin: SF) for biomedical applications such as silk suture, wound dressing, drug delivery systems, and scaffolds [65,66,67,68,69]. Previous studies have shown that the presence of SF in the scaffold could stimulate the proliferation properties of cells [57,70]. Besides, the integrated osteochondral scaffolds from silk fibroin can effectively support cartilage and bone tissue generation in vitro and are potentially applicable for osteochondral tissue engineering in vivo [71]. HA, CS, and SF can be extracted locally from industrial waste (HA: the meat industry, CS: the seafood industry, and SF: silkworm farms and the silk textile industry). Thus, the use of these materials for biomedical applications produces added value to local industrial waste. Because of their properties, PLA, PCL, HA, CS, and SF were selected for use in this study. Three-dimensional printing and lyophilization were carried out to fabricate bilayer’s bioactive-biomaterials scaffold (BBBS) for FTAC treatment. The principal aim of this study was to evaluate the mechanical/physical properties and the biocompatibility of the BBBS as a biomedical strategy potentially useful in tissue engineering for FTAC treatment.

## 2. Materials and Methods

**Figure 1:**
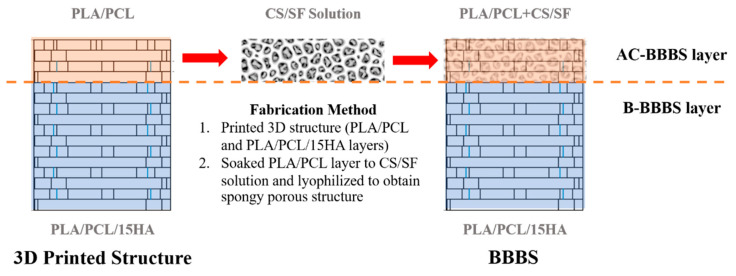
The fabrication diagram of the bilayer’s bioactive-biomaterials scaffold (BBBS). PLA, polylactic acid; PCL, polycaprolactone; CS, chitosan; SF, silk fibroin; HA, hydroxyapatite; AC, articular cartilage.

The BBBS for FTAC treatment was designed based on the osteochondral area of the knee joint femoral condyles, which consists of AC and bone layers. Therefore, the BBBS was developed to plug into the injury site instead of the real osteochondral plug and provide both AC and bone regeneration in the defect area. The AC layer of the scaffold (AC-BBBS layer) provides a proper environment for chondrocyte cell culture before the implantation. Meanwhile, the bone layer of the BBBS (B-BBBS layer) was prepared for bone formation inside the designed structure after being placed in the defect area. The fabrication of BBBS is briefly presented in Figure 1.

### 2.1. Hydroxyapatite Preparation

For HA preparation, bovine bone was cut into small pieces. The tissue and ligament were removed after soaking the bone in H_2_O_2_ (30%, Merck, Darmstadt, Germany) for 2 days. Then, the clean, small pieces of bones were boiled in 100 °C water to eliminate organic substances. Next, the cleaned bones were dried in a 120 °C hot air oven for 7 h to reduce the moisture. The dried bones were calcined at 850 °C for 3 h [52] before being ground by a local custom high-speed ball milling machine until the particle size was less than 20 μm [72].

### 2.2. Silk Fibroin Preparation

SF was extracted from the local Thai *Bombyx mori* (Nang-Noi, Chiang Mai, Thailand) silk cocoons. They were cut into small pieces and degummed in 0.02 M of Na_2_CO_3_ solution (Ajax Finechem Pty Ltd., Auckland, New Zealand) at 90–100 °C for 30 min and rinsed with warm deionized (DI) water. This process removes the sericin, which causes immunogenic reactions. The degummed silk was dried overnight in a 37 °C hot air oven. Then, dried degummed silk was dissolved in ternary solvent (CaCl_2_/C_2_H_5_OH/H_2_O; 1:2:8 in mole ratio) and incubated at 70 °C for 6 h [52]. CaCl_2_ and C_2_H_5_OH were purchased from Ajax Finechem Pty Ltd., Auckland, New Zealand and Merck, Darmstadt, Germany, respectively. The silk-ternary solution was dialyzed in DI water using a cellulose membrane (Dialysis Tubing D9652, Sigma-aldrich, MWCO 12164, St Louis, MO, USA) at 4 °C for 72 h. DI water was changed every 24 h. The dialyzed SF solution was centrifuged at 2000 rpm for 10 min to separate the waste, and frozen at −80 °C. The frozen SF solution was lyophilized to obtain the SF sponges.

### 2.3. Filament Extrusion

**Figure 2:**
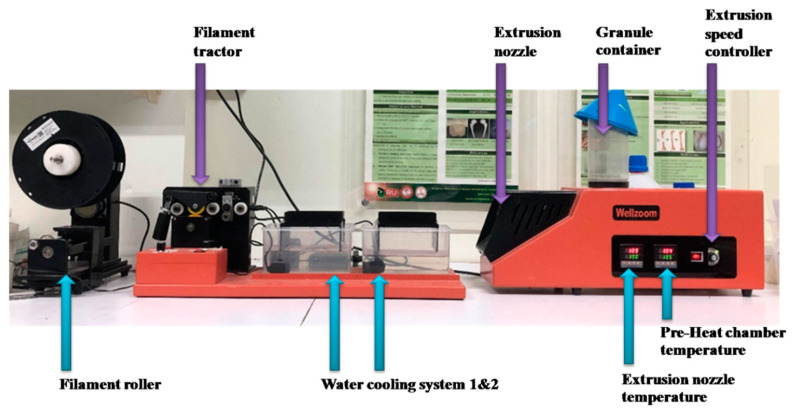
Wellzoom Desktop Extruder Line II with filament cooling system.

Composite biomaterial filaments for 3D printing were locally extruded using a desktop single-screw extruder (Wellzoom Desktop Extruder Line II, Shenzhen Mistar Technology Co., Ltd., Guangdong, China) with the filament cooling system, as shown in Figure 2. The material mixing ratios were obtained from a previous study [15]. The PLA/PCL represented the mixed granules between 70% of PLA and 30% of PCL. The PLA/PCL/15HA represented 85% of PLA/PCL and 15% of HA [15]. PLA (Ingeo 3D850, Natureworks LLC, Blair, Blair, NE, USA) and PCL (Mw 60,000 to 80,000 Da, Daigang Biomaterials, Shandong, China) granules were blended to increase the material’s properties, both biological and mechanical [73,74,75]. The preparation process of these blended materials was modified from a previous study [15]. The PCL and HA were dry mixed in a local custom high-speed ball milling for 12 h. As a result of the low melting temperature of PCL (approximately 60 °C), the softening temperature of PCL is around 40 °C [76]. During the high-speed ball milling process, the heat accumulated up to 45 °C. Therefore, HA powder was thoroughly attached to the softening surface of the PCL granule. The PLA granule was dried in a 60 °C hot air oven for 4 h to reduce the moisture. The dried PLA was immediately added to the ball milling containing PCL + HA at 12 h and the milling process was continued for another 12 h. The PLA/PCL filament was selected for the AC-BBBS layer fabrication, while the PLA/PCL/15HA was selected for the B-BBBS layer. 

Extrusion conditions of both filaments for B-BBBS and AC-BBBS layers were set up similarly. The temperature of the pre-heat chamber was set at 190 °C, while the extrusion nozzle was set in the range of 190–200 °C. After turning the temperature on, the materials were kept in the pre-heat chamber for 15 min before being supplied to the heated nozzle. The first run of the filament was manually tracked to the water-cooling system until the starting tip of the filament reached the filament tractor. Then, the extrusion speed and tractor speed were adjusted until the extruded filament diameter was 1.75 ± 0.05 mm and running smoothly. The filament, produced in the suitable conditions, was rolled to the filament spool using a filament roller, which adjusted the rolling speed related to the filament tractor speed. The diameters of the extruded filaments were measured and the extrusion parameters were adjusted until the extruder provided a constant diameter (1.75 ± 0.05 mm) [77,78]. Then, the filaments were randomly measured during the extrusion. The extruded filaments were randomly selected at 2 m lengths. The diameters of the 2 m selected filaments were measured and recorded every 20 cm.

### 2.4. Hydroxyapatite and Silk Fibroin Characterization

X-ray diffraction (XRD) was used to characterize the phase composition of the extracted HA and the HA within the extruded filaments, while the functional group of SF sponge was characterized using Fourier transform infrared spectroscopy (FTIR: Thermo Fisher Scientific, Waltham, MA, USA).

### 2.5. Mechanical Tests for 3D-Printed Specimen

**Figure 3:**
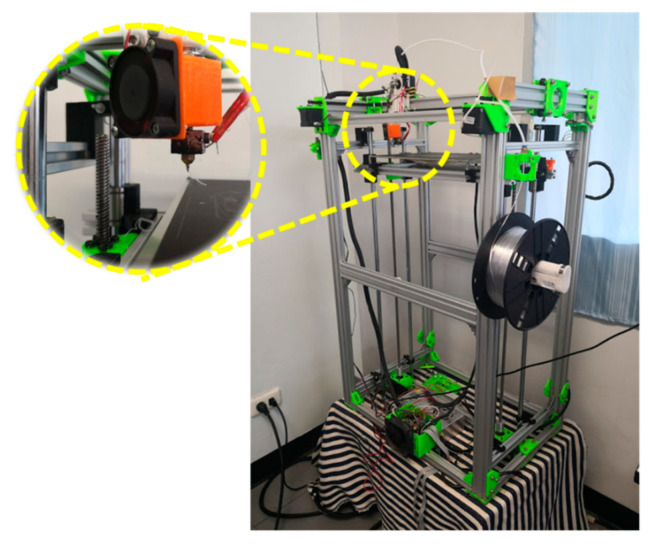
The core XY FFF 3D printer locally made by the Biomedical Engineering Institute (BMEI) laboratory.

**Figure 4:**
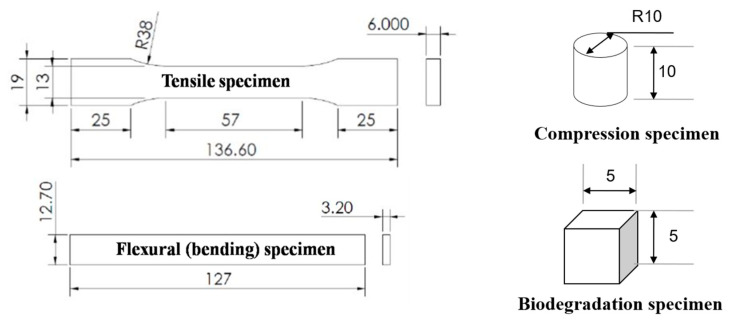
The 3D printer locally made by BMEI laboratory.

Mechanical tests were performed to determine the mechanical properties for finite element analysis. The mechanical test specimens from the extruded filaments were printed using a core XY FFF 3D printer that was made locally by the Biomedical Engineering Institute (BMEI) laboratory, as shown in Figure 3. The mechanical tests consisted of compression, tension, and bending. Each test specimen followed the American Society for Testing and Materials (ASTM) and previous studies for the recommended shape and size. The compression specimen was printed in a cylindrical shape with a 10 mm diameter and 10 mm thickness (Figure 4). The specimen was placed in the compression station of a universal testing machine (UTM). The crosshead motion rate was set at 5 mm/min with a 10 kN loading cell [79]. The tensile specimen was printed in the recommended dimension of the tensile testing standard (ASTM D638), as shown in Figure 4. The specimen was set in the tensile jig fixture of the UTM. The crosshead motion was 5 mm/min with a 100 N loading cell [78]. The bending test was performed on a rectangular-shaped 3D-printed specimen following the ASTM D790 standard (Figure 4). The specimen was placed in the three-point bending testing set of the UTM. The crosshead motion was 10 mm/min with a 5 kN loading cell [78].

### 2.6. Biodegradation Test

The degradation of materials in vitro can be used to estimate their behavior in vivo. Phosphate buffer saline (PBS) solution can be used for the polymer degradation test [80]. However, the biological compound of the body fluid consists of several enzymes [81]. Lysozyme has been found in various human body fluids in different concentrations [82] and it is one of the cheapest enzymes on the biochemical market. Cubic 3D-printed specimens with 5 × 5 × 5 mm^3^ were used for the biodegradation test (Figure 4). At first, the dry weight of each specimen was recorded as Ws. A previous study estimated the mean serum lysozyme concentration in normal adults at 1.6 µg/mL [83]. Therefore, biodegradability was evaluated after soaking the sample in PBS containing 1.6 µg/mL of lysozyme in a 37 °C incubator for 7, 15, and 30 days. The samples were repeatedly rinsed with DI water at the end of the incubation period. The rinsed samples were frozen and lyophilized for 48 h. Finally, the final dry weight of the specimens was recorded as We, and its biodegradation percentage was determined using Equation (1).

Biodegradability (%) = (Ws − We) × 100/(Ws)(1)

### 2.7. 3D Printing Conditions and Scaffold Fabrication

**Figure 5:**
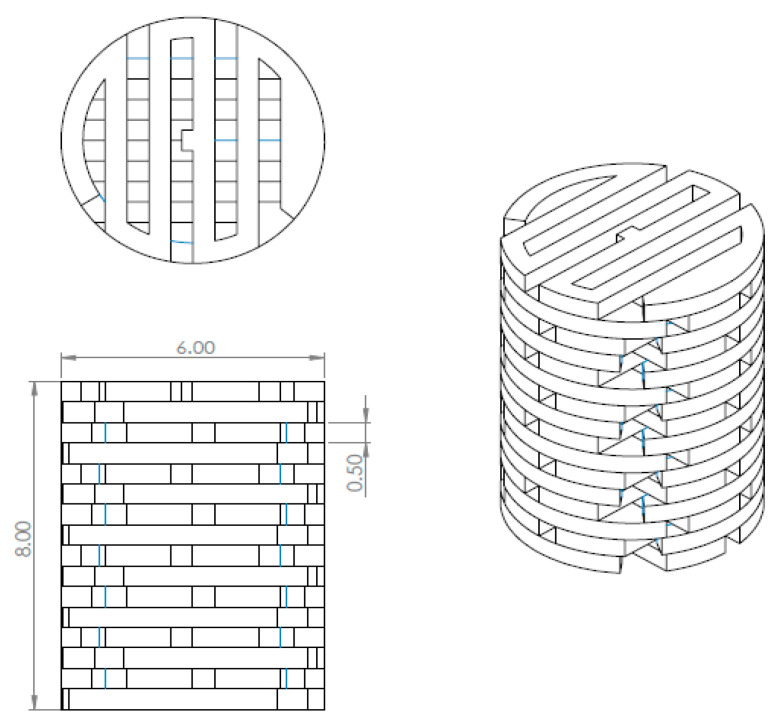
The design and dimensions of total 3D printing model (Unit: mm).

The scaffold was printed using the core XY FFF 3D printer. The single platform was designed to print continuously and be connected to the next platform. Every single platform was rotated 90° against the lower platform. Each platform was 0.5 mm in height and a printed set contained four single platforms. The PLA/PCL/15HA was printed for three sets and the PLA/PCL was printed for the last set. The total height of the 3D-printed structure was 8 mm with a 6 mm diameter, as shown in Figure 5. The nozzle size of the printer was 0.4 mm. The printing conditions consisted of a 0.2 mm printed layer height, and a nozzle melting temperature of 200 °C.

After the 3D structure fabrication was completed, the CS/SF solution was prepared for lyophilization. CS flakes derived from a squid pen with 94.69% deacetylation degree (Taming Enterprise co., Ltd., Samutsakon, Thailand) were cut into small pieces and soaked in 1% (v/v) acetic acid solution (100% acetic acid, Merck, Darmstadt, Germany) in the ratio of 0.5 g/100 mL, until completely dissolved (approximately 3–5 days). Then, 0.5 g of SF sponge [15,57,58,84] was added to the CS solution and homogeneously mixed. The 25% glutaraldehyde (GA: 25% in water, Merck, Darmstadt, Germany) was diluted in DI water (1/100, v/v) and used as a cross-linking agent. Thereafter, 100 mL of CS/SF solution was mixed with 1 mL of the diluted GA solution before combining with the 3D-printed structure. The CS/SF solution was filled in 96-well plates (flat bottom) at 150 µL/well. Then, the 3D printing structure (PLA/PCL layer) was inserted into the well plate and frozen at −80 °C freezer overnight. The frozen BBBS was lyophilized for 48 h. The final BBBS consists of the AC-BBBS (PLA/PCL + CS/SF) and B-BBBS (PLA/PCL/15HA), as shown in Figure 1.

### 2.8. Scanning Electron Microscope (SEM) Observation

The morphology, outer surface, and inside structure of the BBBS were observed under SEM (JEOL JSM 6400, Tokyo, Japan).

### 2.9. Cell Culture and Cell Viability Test

Cell culture for AC-BBBS layer: The human chondrosarcoma cell line, SW1353 chondrocyte-like cells (ATCC® HTB-94™), were expanded in a T75 culture flask using 15 mL of the complete Dulbecco’s modified Eagles medium (DMEM, Gibco®, Gaithersburg, MD, USA) with 10% fetal bovine serum (FBS, Gibco®, Gaithersburg, MD, USA) and 1% of Anti-Anti (penicillin: 10,000 units/mL, streptomycin: 10,000 µg/mL, and Gibco Amphotericin B: 25 µg/mL, Gibco®, Gaithersburg, MD, USA) until reaching cell confluence. The specimens were placed in 24-well plates. Then, 500 µL of the complete DMEM, which contained 5000 cells of SW1353, was directly seeded on the surface of each specimen and the specimens were allowed to absorb the cells containing DMEM for approximately 10 min. After that, another 500 µL of the complete DMEM was filled in the well plate and incubated at 37 °C under 5% CO_2_.

Cell culture for the B-BBBS layer: The human fetal osteoblast cell line, hFOB1.19 (ATCC® CRL-11372™), was expanded in a T75 culture flask using 15 mL of complete DMEM/F-12: DMEM/Ham’s F-12 medium (phenol red free, Gibco®, Gaithersburg, MD, USA) supplemented with 10% FBS and 1% Anti-Anti (penicillin: 10,000 units/mL, streptomycin: 10,000 µg/mL, and Gibco Amphotericin B: 25 µg/mL, Gibco®, Gaithersburg, MD, USA) until reaching cell confluence. The B-BBBS layer specimens were placed in 24-well plates. Then, 500 µL of complete DMEM/F-12, which contained 5000 cells of hFOB1.19, was directly seeded on the surface of each specimen and the specimens were allowed to absorb the cells containing DMEM/F-12 for approximately 10 min. After that, another 500 µL of complete DMEM/F-12 was filled in the well plate and incubated at 37 °C under 5% CO_2_.

Cell viability test using MTT assay: MTT solution was prepared before the test. Phosphate buffer saline (PBS tablets, AMRESCO, Inc., Solon, OH, USA) was dissolved in 100 mL of DI water, and 10 mL of PBS solution was mixed with 50 mg of MTT powder (3-(4,5-Dimethylthiazolyl-2)-2,5-diphenyl tetrazolium bromide, AMRESCO, Inc., Solon, OH, USA). Then, the MTT solution was filtered using a 0.22 µm nylon syringe filter. The MTT solution was added to the testing well plate containing culture medium with the dilution 1:10 (MTT: culture medium). Then, the testing well plate was incubated in a CO_2_ incubator at 37 °C for 4 h. After the incubation, the solution in the testing well plate was removed and the dimethyl sulfoxide (DMSO, Merck, Tokyo, Japan) solution was filled at 2 mL/well. The testing well plate was shaken using a vertical shaker for 20 min. Finally, the optical density (OD) of the solution of each well was measured using a spectrophotometer. The ODs were recorded, and the cell viability was calculated using the following equation. The average OD of the control group on day 1 was assumed as healthy cells with 100% viability (OD_C1_). The OD of the other groups and culture duration (OD_GD_) were calculated individually using Equation (2). The final cell viability percentage of each group was presented as the average value.

Cell viability (%) = (OD_GD_) × 100/(OD_C1_) (2)

Cell viability test for AC-BBBS layer: There were three cell culture durations consisting of 1, 7, and 14 days.

On day 1, the MTT assay was performed on the 1-day group, consisting of the control, PLA/PCL, and PLA/PCL + CS/SF. The control represented the only cell culture without the testing specimen. The PLA/PCL represented the cell culture with the 3D-printed PLA/PCL specimen. The PLA/PCL + CS/SF represented the cell culture with the AC-BBBS layer specimen. According to the morphology of our scaffold, the seeded cells had fallen as a result of gravity, passing through the porous cavities to the bottom of the well plate. Therefore, the cells were attached on each layer of the specimen and on the well plate. To investigate only the cells attached on the specimens, the PLA/PCL and PLA/PCL + CS/SF specimens of the 7- and 14-day groups were moved from the initial well plate to a new well plate. Thus, only cells attached on the specimen were continuously cultured in the new well plate until day 7 and 14. The leftover cells in the initial well plate of the 7-day group (on day 1) were selected to perform the MTT assay, and the cell viability percentages of these leftover cells were used to calculate and estimate the percentage of cell attached on the specimen of the 1-day group. Therefore, the cell viability percentages on day 1 were presented in five groups consisting of the control, PLA/PCL, PLA/PCL + CS/SF, cells attached on the PLA/PCL specimens (cell attached-PLA/PCL), and cells attached on the PLA/PCL + CS/SF specimens (cell attached-PLA/PCL + CS/SF) groups.

**Figure 6:**
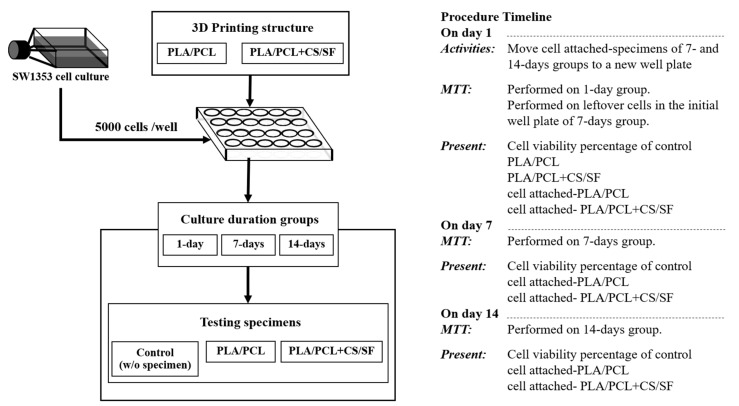
The experimental flowchart of cell culture for the AC layer of the scaffold (AC-BBBS layer).

On day 7 and day 14, the MTT assays were performed and the cell viability percentages were presented in three groups consisting of the control, cell attached-PLA/PCL, and cell attached-PLA/PCL + CS/SF groups, as shown in Figure 6.

Cell viability test for B-BBBS layer: There were three cell culture durations consisting of 1, 7, and 14 days.

On day 1, the MTT assay was performed on the 1-day group, consisting of the control, PLA/PCL, and PLA/PCL/15HA. The control represented the only cell culture without the testing specimen. The PLA/PCL represented the cell culture with the 3D-printed PLA/PCL specimen. The PLA/PCL/15HA represented the cell culture with the B-BBBS layer specimen. According to the morphology of our scaffold, the seeded cells had fallen as a result of gravity, passing through the porous cavities to the bottom of the well plate. Therefore, cells were attached on each layer of specimen and on the well plate. To only investigate cells attached on the specimens, the PLA/PCL and PLA/PCL/15HA specimens of the 7- and 14-day groups were moved from the initial well plate to a new well plate. Thus, only cells attached on the specimen were continuously cultured in the new well plate until day 7 and 14. The leftover cells in the initial well plate of the 7-day group (on day 1) were selected to perform the MTT assay and the cell viability percentages of this leftover cells were used to calculate and estimate the percentage of cells attached on the specimen of the 1-day group. Therefore, the cell viability percentages on day 1 were presented in five groups consisting of the control, PLA/PCL, PLA/PCL/15HA, cells attached on the PLA/PCL specimens (cell attached-PLA/PCL), and cells attached on the PLA/PCL + CS/SF specimens (cell attached-PLA/PCL/15HA) groups.

**Figure 7:**
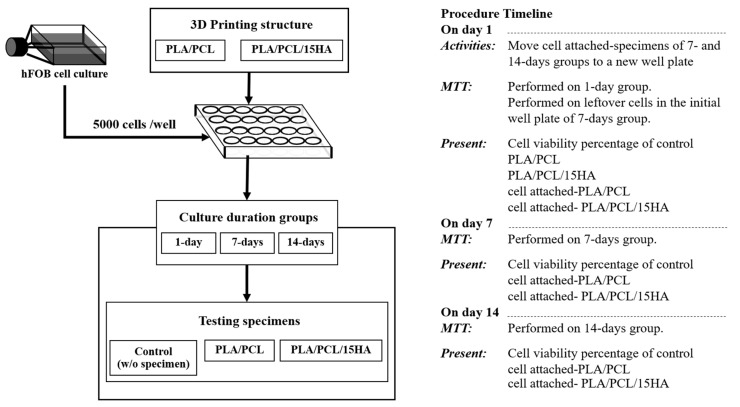
The experimental flowchart of cell culture for the bone layer of the scaffold (B-BBBS layer).

On day 7 and day 14, the MTT assays were performed and the cell viability percentages were presented in three groups consisting of the control, cell attached-PLA/PCL, and cell attached- PLA/PCL/15HA groups, as shown in Figure 7.

### 2.10. Finite Element Analysis

For finite element analysis (FEA), the mechanical properties were obtained from the mechanical tests. The simulating software ANSYS R15.0 Workbench (ANSYS, Inc., Canonsburg, PA, USA) was used to analyze the maximum load that the designed structure could carry. The base of the model was fixed, and the distributed compression load was applied to the surface of the model. The applied load was randomly applied until the maximum principle stress of the model reached the minimum mechanical strength of the 3D-printed materials. The AC-BBBS thickness for FEA was 2 mm, which was selected from the normal thickness of the AC at the femoral condyles (approximately 1.65 to 2.65 mm) [85]. The mechanical properties of PLA/PCL were applied to the AC-BBBS layer. Meanwhile, the mechanical properties of PLA/PCL/15HA were applied to another 6 mm of the structure (the B-BBBS layer) for the FEA.

## 3. Results

### 3.1. XRD

**Figure 8:**
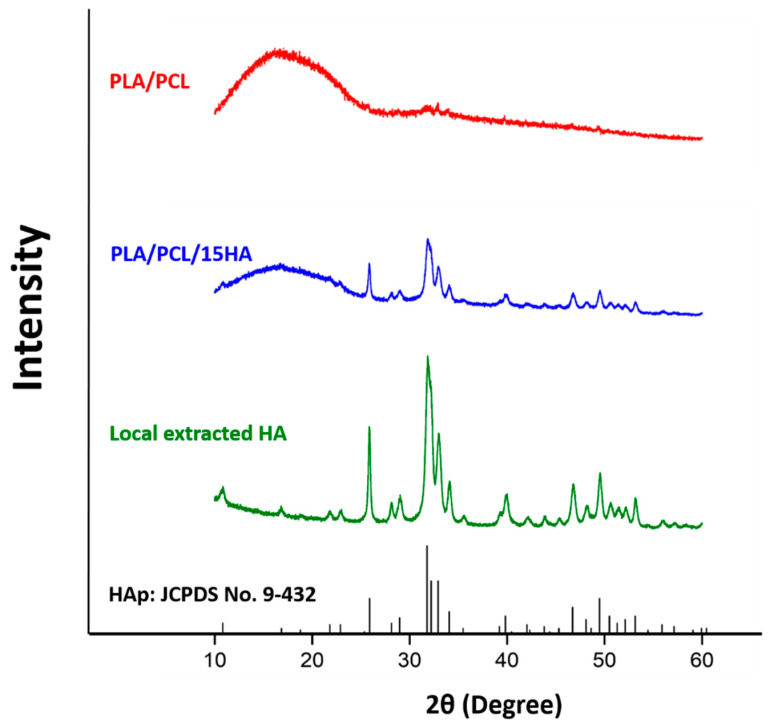
X-ray diffraction (XRD) patterns of polylactic acid (PLA)/polycaprolactone (PCL), PLA/PCL/15HA, and local extracted HA show that the local extracted HA and its presence in PLA/PCL/15HA are correspond to the characteristic peak of hydroxyapatite (HAp) (Joint Committee on Powder Diffraction Standards file number (JCPDS) No. 9-432).

The XRD patterns of local extracted HA, PLA/PCL, and PLA/PCL/15HA samples are presented in Figure 8 within the 2θ range 10° to 60°. The resolved XRD peaks of the local extracted HA and those of HA in PLA/PCL/15HA are shown within the 2θ range 20° to 50°. The sharp diffraction peak positions of pure crystalline HA were observed and are presented in Table 1. The 2θ values of the local extracted HA: 25.86°, 31.86°, 32.2°, 32.97°, and 39.99° correspond to the diffraction planes (h k l values) at (0 0 2), (2 1 1), (1 1 2), (3 0 0), and (3 1 0), respectively. These main characteristic peaks of the local extracted HA are similar to the Joint Committee on Powder Diffraction Standards (JCPDS) reference standard file number 09-0432. Besides, the broad peak of the PLA/PCL blend component is located between 10.00° and 25.00°. The composites reveal peaks for both HA and PCL/PLA. The presence of the crystalline HA phase incorporated in the polymer matrix appears at almost the same peak positions compared with the local extracted HA sample. This characteristic indicates that the dispersion of HA in the matrix is very uniform. Nonetheless, their intensity is weakened because of the low content of HA incorporated in the matrix.

### 3.2. FTIR

**Figure 9:**
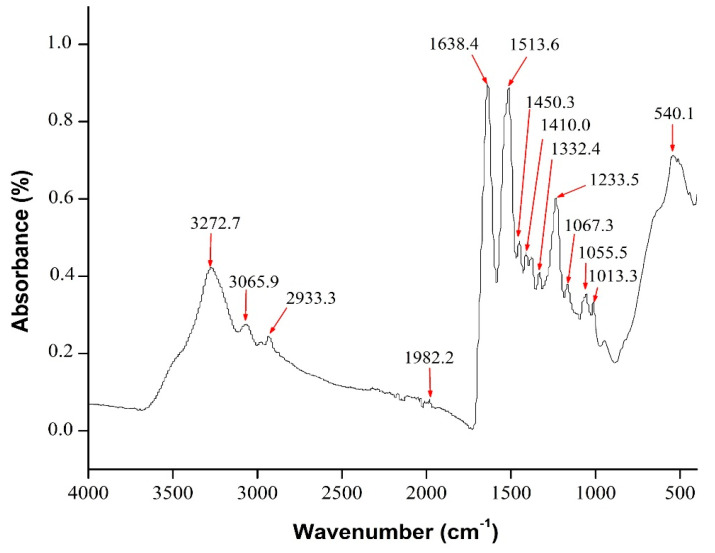
Fourier transform infrared spectroscopy (FTIR) spectrum of the local extracted SF.

The extraction of local Thai *Bombyx mori* (Nang-Noi) SF was characterized using FTIR. The random coils (silk I) and β-sheet (silk II) are major conformations of the *Bombyx mori* SF structure. The FTIR spectrum of the local extracted SF is shown in Figure 9. The FTIR spectrum of the local extracted SF was related to the recommended wavenumbers, as presented in Table 2 [86]. The spectrum was also similar to previous studies with the same extraction technique [15,86]. The FTIR peaks at 1637.7 cm^−1^, 1514.3 cm^−1^, and 1234.1 cm^−1^ represent amide I, amide II, and amide III, respectively.

### 3.3. Compression Test

**Figure 10:**
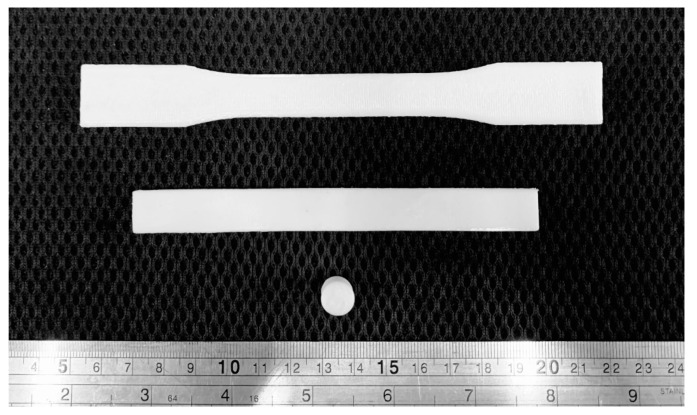
Specimen for the tensile test, bending test, and compression test (from top).

The compression tests were performed on the cylindrical specimens (Figure 10). The summary of the compression testing results, consisting of the ultimate strain, ultimate stress, and modulus of elasticity, is shown in Table 3. A significantly higher compressive stress was demonstrated by the PLA/PCL/15HA specimen with 83.19 ± 1.63 MPa, compared with the PLA/PCL specimen.

### 3.4. Tensile Test

The tensile specimen is shown in Figure 10. Table 4 is the summary of all the mechanical properties that result from the tensile test. The table summarizes the average elongation at break, ultimate stress, and modulus of elasticity of the specimens from each material’s composition. The PLA/PCL specimen provided the highest values of ultimate stress and modulus of elasticity, at 64.29 ± 3.64 MPa and 1.10 ± 0.03 GPa, respectively. The lowest ultimate stress, by a significant amount, was shown by the PLA/PCL/15 specimen (52.91 ± 1.73 MPa) compared with the PLA/PCL specimens. The addition of HA in the composite materials resulted in the lowest tensile stress.

### 3.5. Flexural (Bending) Test

The rectangular specimen, as shown in Figure 10, of both material compositions was used in flexural (bending) testing. Table 5 compiles the average and standard division results for both specimen compositions. There was no difference between the PLA/PCL and PLA/PCL/15HA printed specimens.

### 3.6. Biodegradation Test

**Figure 11:**
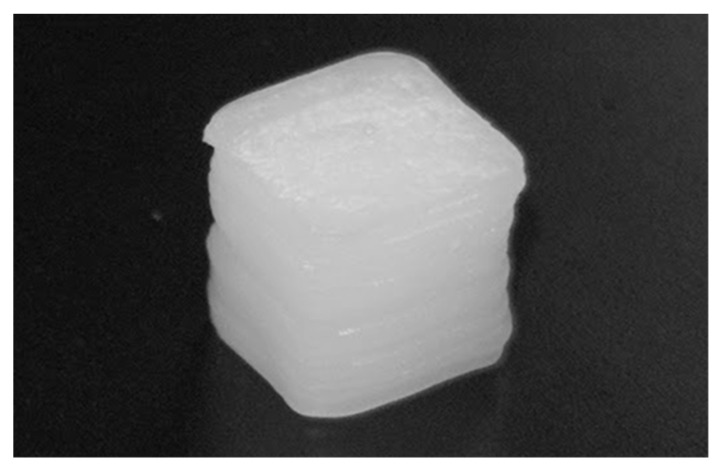
Close up image of 3D-printed specimen for biodegradation test.

**Figure 12:**
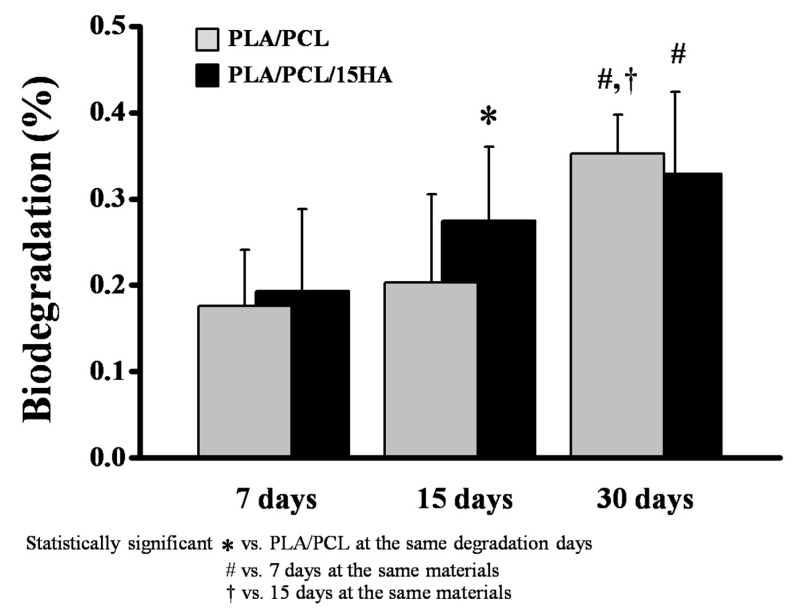
Biodegradation percentage.

The biodegradation test was performed on the cubic printed specimens, as shown in Figure 11. The weight difference of the specimens was calculated and presented in biodegradation percentage, as shown in Figure 12. After soaking the specimens in PBS solution containing lysozyme for 7, 15, and 30 days, the biodegradation percentage was obtained after comparing the collected data according to degradation days with the data of the starting point. The degradation percentages are presented as mean ± SD. The comparison between the degradation days of the same material compositions presented a slight increase in the biodegradation percentage, related to the increasing number of degradation days. However, a statistical difference was seen on day 30 for both groups. On day 30, PLA/PCL presented the highest degradation percentage (0.35 ± 0.05%), by a significant amount, as compared with the same group on days 7 and 15. Furthermore, the PLA/PCL/15HA groups presented the highest degradation percentage (0.33 ± 0.09%) on day 30, significantly higher than day 7 only. When comparing between the PLA/PCL and PLA/PCL/15HA groups on day 15, the biodegradation percentage of PLA/PCL/15HA (0.27 ± 0.09%) was significantly higher than that of PLA/PCL (0.2 ± 0.1%). The dimensions of the specimens were also investigated, but there was no change in the dimensions after 30 days of degradation.

### 3.7. BBBS Morphology

**Figure 13:**
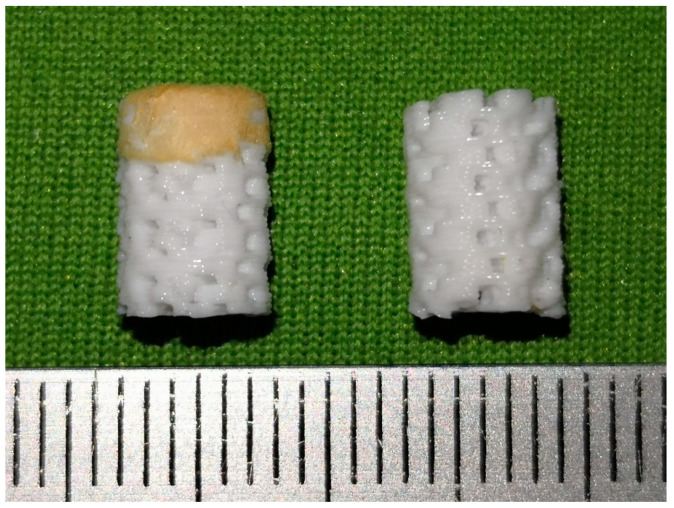
Overall morphology of bilayer’s bioactive-biomaterials scaffold (BBBS). (right: 3D-printed structure only) (left: 3D-printed structure with lyophilized chitosan (CS)/SF)).

The bilayer 3D printing structure was printed into the AC-BBBS and B-BBBS layers using PLA/PCL and PLA/PCL/15HA filaments, respectively. Then, the AC-BBBS layer was combined with the CS/SF solution and lyophilized, as shown in Figure 13.

**Figure 14:**
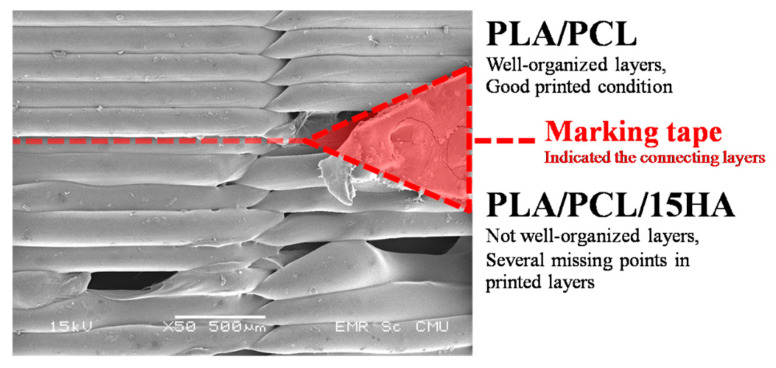
The morphology of printed layers.

The structure and size of BBBS were designed based on the requirements of the consultant orthopedic surgeons (Department of Orthopedics, Faculty of Medicine, Chiang Mai University, Chiang Mai, Thailand). The difference between PLA/PCL and PLA/PCL/15HA layers was not easily distinguishable because of the same color of the raw material granules. However, it can be seen at certain tilted angles. The different morphology of the printed structure between PLA/PCL and PLA/PCL/15HA filaments was observed using SEM. Masking tape was placed to indicate the connecting layer between PLA/PCL and PLA/PCL/15HA, as shown in Figure 14. The layer of PLA/PCL was better organized than the PLA/PCL/15HA printed layer using the same printing conditions.

**Figure 15:**
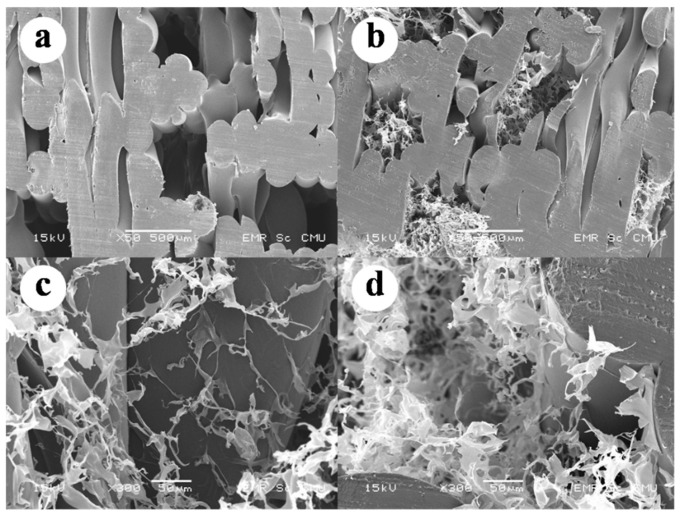
Scanning electron microscope (SEM) images of bilayer biomaterial cell scaffold: (**a**) free cavities inside 3D-printed structure, (**b**) CS/SF porous structure filled the cavities of 3D-printed structure, (**c**,**d**) CS/SF structure attached to the surface of the 3D-printed structure.

After combining the 3D-printed structure to the CS/SF (Figure 13), the morphology of the completed bilayer biomaterial cell scaffold was analyzed using SEM. The scaffold was cut in half longitudinally, using a plain cutter, to observe the inside structure. The connection between each layer was homogeneously melted, as shown in Figure 15a,b. In the AC-BBBS layer, the CS/SF solution thoroughly filled the cavities in the 3D-printed structure, as shown in Figure 15b. The porous structure of the CS/SF was distributed in the cavity and connected to the surface of the 3D-printed structure, as shown in Figure 15c,d. Therefore, the interconnected porous CS/SF was thoroughly distributed in the AC-BBBS layer.

### 3.8. Cell Viability Test for AC-BBBS

**Figure 16:**
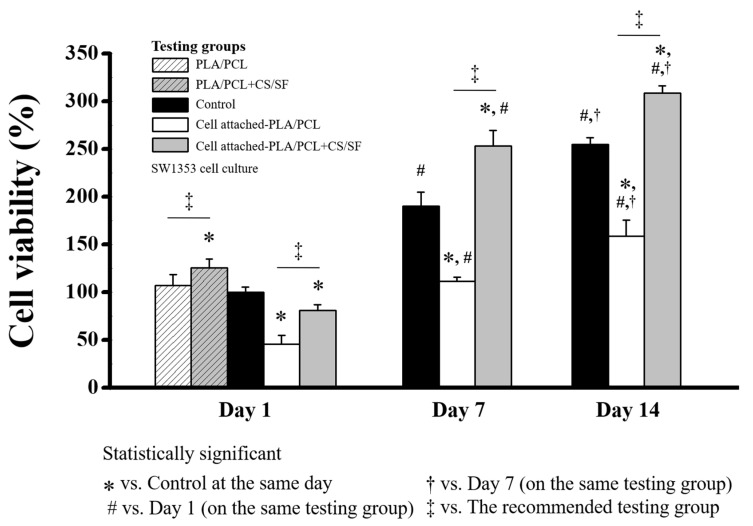
The cell viability percentage of the scaffold (AC-BBBS layer), *p*-value < 0.05.

The MTT assay was performed on the scaffold with SW1353 cells and the cell viability percentage is presented in Figure 16. On day 1, the MTT assay was performed, and the cell viability percentages of the five testing groups, consisting of the control, PLA/PCL, PLA/PCL + CS/SF, cell attached-PLA/PCL, and cell attached-PLA/PCL + CS/SF, are presented in Figure 16. The PLA/PCL + CS/SF group presented the highest cell viability percentage (125.25 ± 9.36%), by a significant margin. The cell viability percentages of PLA/PCL, cell attached-PLA/PCL, and cell attached-PLA/PCL + CS/SF were 106.79 ± 11.67%, 45.84 ± 8.72%, and 80.99 ± 6.11%, respectively. The PLA/PCL was the only group that showed no difference from the control group. The comparison between cell attached-PLA/PCL and cell attached-PLA/PCL + CS/SF indicated that the cell attachment ability of the PLA/PCL + CS/SF specimen was higher than that of the PLA/PCL specimen.

On day 7, the cell viability of the control, cell attached-PLA/PCL, and cell attached-PLA/PCL + CS/SF presented as 190.29 ± 14.49%, 111.17 ± 4.65%, and 252.92 ± 16.18%, respectively. On this day, the cell viability percentages of all the groups were significantly higher than the same groups tested on day 1. The cell viability percentage of the cell attached-PLA/PCL group was still significantly lower than the control group, while that of the cell attached-PLA/PCL + CS/SF was significantly higher than the control group.

On day 14, the cell viability percentage of cell attached-PLA/PCL + CS/SF dramatically increased (308.28 ± 7.88%) and was significantly higher than the control and the cell attached-PLA/PCL groups (254.56 ± 7.7% and 158.62 ± 16.61%). These results indicate the cell proliferation ability of both scaffolds (PLA/PCL and PLA/PCL + CS/SF), especially in the PLA/PCL + CS/SF group. The cell viability percentages of all groups were significantly higher than the same testing groups on days 1 and 7.

### 3.9. Cell Viability Test for B-BBBS

**Figure 17:**
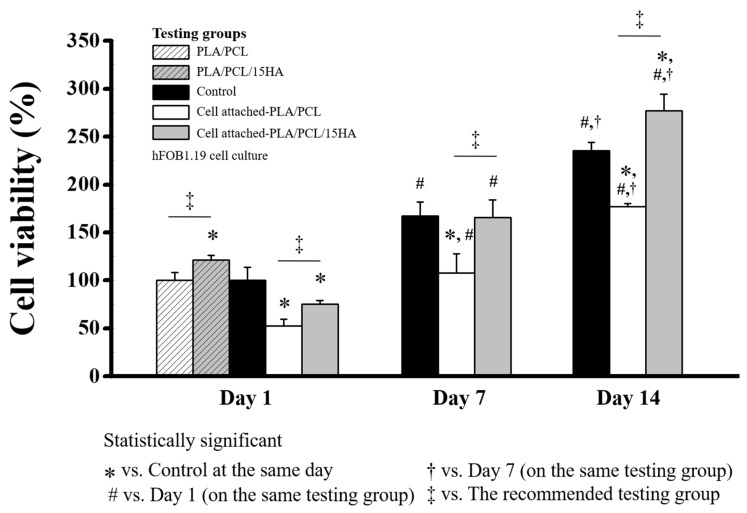
The cell viability percentage of the scaffold (B-BBBS layer), *p*-value < 0.05.

The results are presented in Figure 17. The cell viability trend of the B-BBBS layer was in the same direction as the AC-BBBS layer. On day 1, the cell viability percentages of the control, PLA/PCL, PLA/PCL + CS/SF, cell attached-PLA/PCL, and cell attached-PLA/PCL/15HA were 100.00 ± 1.60%, 100.30 ± 8.07%, 121.13 ± 4.98%, 52.87 ± 6.92%, and 75.17 ± 4.15%, respectively. The comparison between the control group and the other groups showed that only PLA/PCL had no difference. On the other hand, PLA/PCL/15HA presented the highest cell viability percentage, by a significant margin. In this layer, cells were attached to the PLA/PCL/15HA specimen more than the PLA/PCL specimen, the same as the result of the PLA/PCL + CS/SF specimen in the AC-BBBS layer.

On day 7, the cell viability percentages of the control, cell attached-PLA/PCL, and cell attached-PLA/PCL/15HA were 167.44 ± 14.43%, 108.08 ± 19.61%, and 165.69 ± 18.55%, respectively. All groups presented a significantly higher cell viability percentage compared with day 1. From being significantly lower than the control group on day 1, the cell viability percentage of cell attached-PLA/PCL/15HA showed no difference from the control group on day 7.

On day 14, the cell viability percentages of the control, cell attached-PLA/PCL, and cell attached-PLA/PCL/15HA were 235.39 ± 8.51%, 177.18 ± 2.87%, and 277.21 ± 16.93%, respectively. The cell viability percentage of cell attached-PLA/PCL/15HA was dramatically increased and significantly higher than the control and cell attached-PLA/PCL groups.

### 3.10. Finite Element Analysis

**Figure 18:**
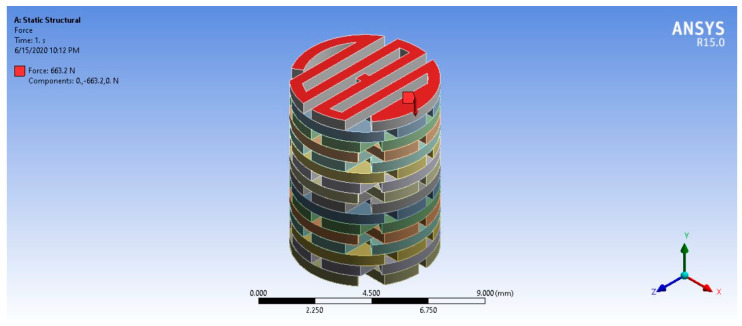
The finite element analysis (FEA) model using the simulating software ANSYS.

**Figure 19:**
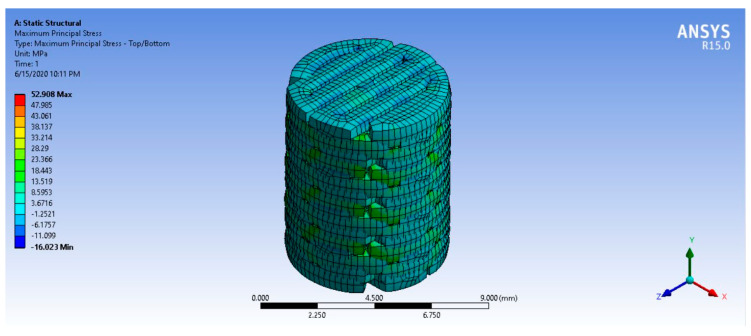
FEA simulation result: the principal stress of the 663.2 N applied load.

**Figure 20:**
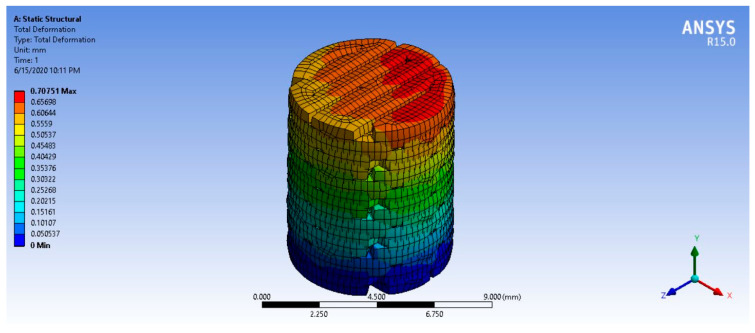
FEA simulation result: the total deformation of the 663.2 N applied load.

The mechanical properties of PLA/PCL and PLA/PCL/15HA were obtained from the mechanical tests and applied to the FEA program. The lowest mechanical strength of the fabricated BBBS was the tensile strength of PLA/PCL/15HA (52.91 ± 1.73 MPa). The base of the 3D model was fixed, and the distributed compression load was randomly applied to the surface of the model (on the surface of the AC-BBBS layer), as shown in Figure 18. After applying a series of compression loads to the surface of the 3D model, the 663.2 N compression load presented the maximum stress at 52.908 MPa (Figure 19), which reached the lowest mechanical strength of the fabricated BBBS. Therefore, the maximum load should not be over 663.2 N or 66.32 kg. Moreover, the maximum deformation for the 663.2 N applied load was 0.71 mm, as shown in Figure 20.

## 4. Discussion

The characteristics of the extracted bioactive materials (HA and SF) were the same as their standard characteristics (XRD and FTIR patterns). Thus, these extraction methods can be used to extract HA and SF from industrial waste. Furthermore, the pathogens present in bovine bone are removed at temperature above 800 °C [87,88]. Therefore, locally extracted HA has a low possibility of transmitting diseases.

From the SEM observation, the local extruded filaments can be printed into the designed 3D structure. In terms of 3D printing morphology, the PLA/PCL filament was well organized and produced perfect layers. Meanwhile, for the PLA/PCL/15HA, the SEM observations showed that the 3D printing was inconsistent in some layers. However, the PLA/PCL/15HA printed layers kept the overall morphology of the design structure, but could not be distinguished without a microscope.

**Figure 21:**
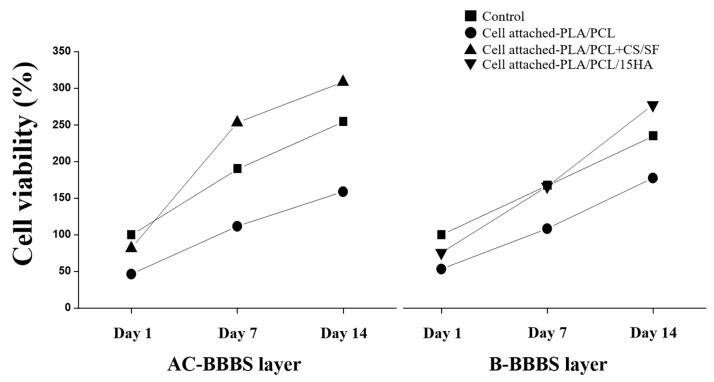
The progression of cell viability percentage in the AC-BBBS and B-BBBS layers.

The cell viability percentage of scaffolds from the AC-BBBS and B-BBBS layers can be plotted as a progression chart, as shown in Figure 21. The presence of bioactive materials in the 3D-printed structure significantly increased the cell viability percentage. The increasing cell viability percentage represented cell proliferation. Therefore, the presence of bioactive materials also increased cell proliferation. The slope of each line graph in Figure 21 shows the proliferation rates. In the AC-BBBS layer, the PLA/PCL + CS/SF scaffold presented the highest slope from day 1 to day 7, which slightly increased from day 7 to day 14. Meanwhile, the cell viability percentages of the control and the cell attached-PLA/PCL were slightly increased from day 1 to day 14. Because of the similar structure of CS and GAGs, there are several studies regarding the fabricated CS scaffold for cartilage regeneration [89,90,91]. CS can provide a good structure for cartilage cell adhesion, and other bioactive materials or growth factors can be added to encourage cell proliferation [57,92,93]. In this study, SF was added to increase the cell proliferation ability. Previous studies have reported that the addition of SF provides better adhesion, growth, and differentiation of chondrocyte cells [94,95,96]. Thus, the presence of CS with SF was the main factor increasing the cell viability percentage in the AC-BBBS layer.

Besides, the B-BBBS layer scaffold containing HA, which had the highest slope as shown in Figure 21, presented the highest proliferation rate. The cell viability percentages of the control and the cell attached-PLA/PCL were slightly increased from day 1 to day 14, the same as in the AC-BBBS layer. Previous studies also reported the ability of commercial HA to promote cell proliferation [97,98]. Therefore, our local extracted HA is the key factor for cell proliferation in the B-BBBS layer.

The degradation of PLA/PCL and PLA/PCL/15HA specimens at the end of the experiment (day 30) presented no difference, and the degradation percentages were only 0.35 ± 0.05% (PLA/PCL) and 0.33 ± 0.09% (PLA/PCL/15HA). From a previous study, the extracellular matrix formation of human articular chondrocyte was completely filled in the scaffold at the end of 21 days of cultivation [99]. Therefore, during the cultivation period, the BBBS still provided structure for cell adhesion, which lasted until the implantation phase. However, the mechanical properties of the BBBS during the longer degradation period should be investigated to estimate their implantation conditions.

The mechanical properties of the 3D-printed specimens obtained from the mechanical tests were similar to our previous study [15]. In terms of scaffold for osteochondral regeneration, the mechanical properties of the scaffold should be related to those of natural AC and bone. Previous studies mention the lubrication properties of the scaffold, which are responsible for the efficient lubrication of AC [100,101]. However, there is no human-made material that can match the articulate friction coefficient of major synovial joints such as hips and knees [102]. Thus, in future studies, the aim of the AC chondrocyte generating AC tissue on the AC-BBBS before implantation in order to provide a suitable lubrication for the joint is of great importance. Moreover, the B-BBBS aim to press-fit into the defect area and let blood flow and clot inside the B-BBBS. The clot release growth factors and cytokines before being absorbed and replaced with newly blood vessel [103]. Thus, natural tissue regeneration occurs in the degraded cavities of BBBS over time.

The FEA was performed to estimate and predict the abilities of the BBBS to carry the compression load. According to the OAT application, single or multiple bone plugs with intact AC were implanted into the defect area [6,9]. During daily activities, the mechanical load was applied to the surface of the knee and the plugs. In the case of a small implantation area, the mechanical load may be distributed to the surrounding native tissue more than the plugs. If the contact area of the plugs reaches the critical point, the mechanical load will be distributed on the plugs in the same way as in the surrounding native tissue. The compression load at the contact area randomly depends on the contact angle [104]. Therefore, a dynamic FEA should be performed before surgery using the patient’s data, such as the size of the defect area and the estimated number of BBBSs, to predict the ability of the BBBS to carry the load. Furthermore, the BBBS thickness can be adjusted onsite using an ordinary knife or cutter. The BBBS shape and size can also be customized using a 3D program.

## 5. Conclusions

From our findings, the BBBS for FTAC treatment demonstrated a high feasibility in terms of animal implantation tests and clinical trials in the near future. The materials we tested from industrial waste provided good biological properties compared with several studies in biomaterials for biomedical and tissue engineering applications. Moreover, the 3D design can be customized to suit the defect area of each patient with a high fabrication speed. Furthermore, 3D printers are now cheaper and easier to access and use, hence treatments will be delivered sooner and at a reasonable price to poor patients in remote areas. Further investigations, such as biomechanical tests, chemical releasing tests, the BBBS biodegradation/mechanical relation, immunohistochemistry, and in vivo studies, must be performed to investigate the effects of the implantation scaffold on the living body.

## Figures and Tables

**Table 1:** The X-ray diffraction (XRD) peaks position of pure hydroxyapatite (HAp) the Joint Committee on Powder Diffraction Standards file number 09-0432 (JCPDS 9-432) and local extracted hydroxyapatite (HA).

2θ: HAp (JCPDS 9-432)	Plane			2θ: HA (Local Extracted)
	h	k	l	
21.82	2	0	0	21.85
22.902	1	1	1	22.99
25.354	2	0	1	25.47
25.879	0	0	2	25.86
28.127	1	0	2	28.14
28.967	2	1	0	28.97
31.774	2	1	1	31.86
32.197	1	1	2	32.2
32.902	3	0	0	32.97
34.049	2	0	2	34.09
35.481	3	0	1	35.49
39.205	2	1	2	39.27
39.819	3	1	0	39.99
42.03	3	1	1	42.09
43.805	1	1	3	43.8
45.306	2	0	3	45.3
46.713	2	2	2	46.75
48.104	3	1	2	48.14
48.624	3	2	0	48.68
49.469	2	1	3	49.54

**Table 2:** Amide wave number and protein secondary structure of silk firoin (SF) [86].

Conformation	Amides and Wavenumbers (cm^−1^)		
	I (CO Stretch)	II (NH Deformation)	III (CN Stretch, NH Bends)
β-sheet	1625–1640	1520–1530	1219–1245
Random coil	1625–1660	1520–1545	1257–1258

**Table 3:** Summary results of compression test. HA, hydroxyapatite.

Specimen Compositions	Mechanical Properties	Mean	SD
PLA/PCL	Ultimate Strain (%)	9.97	1.07
	Ultimate Stress (MPa)	77.92	2.40
	Modulus of Elasticity (GPa)	1.01	0.04
PLA/PCL/15HA	Ultimate Strain (%)	10.68	1.43
	Ultimate Stress (MPa)	83.19*	1.63
	Modulus of Elasticity (GPa)	1.07	0.16

* Statistically significant vs. polylactic acid (PLA)/polycaprolactone (PCL).

**Table 4:** Summary results of tensile test.

Specimen Compositions	Mechanical Properties	Mean	SD
PLA/PCL	Elongation at Break (%)	5.73	1.01
	Ultimate Stress (MPa)	64.29	3.64
	Modulus of Elasticity (GPa)	1.10	0.03
PLA/PCL/15HA	Elongation at Break (%)	5.58	0.45
	Ultimate Stress (MPa)	52.91*	1.73
	Modulus of Elasticity (GPa)	0.97	0.11

* Statistically significant vs. PLA/PCL.

**Table 5:** Summary of flexural (bending) test.

Specimen Compositions	Mechanical Properties	Mean	SD
PLA/PCL	Ultimate Strain (%)	11.94	1.10
	Ultimate Stress (MPa)	104.02	2.12
	Modulus of Elasticity (GPa)	2.24	0.21
PLA/PCL/15HA	Ultimate Strain (%)	11.22	1.69
	Ultimate Stress (MPa)	102.77	3.82
	Modulus of Elasticity (GPa)	2.41	0.40
